# Supplementary material for: Pyruvate Dehydrogenase Kinase 1 inhibition mediated oxidative phosphorylation enhancement in cartilage promotes osteoarthritis progression
Source: BMC Musculoskelet Disord. 2023 Jul 20;24:597. doi: 10.1186/s12891-023-06585-6 (PMC10357736; doi:10.1186/s12891-023-06585-6)
Supplement: Supplementary file 1 — Additional file 1. [file 12891_2023_6585_MOESM1_ESM.zip › MF.Gsea.1653627316242/GOMF_OXIDOREDUCTION_DRIVEN_ACTIVE_TRANSMEMBRANE_TRANSPORTER_ACTIVITY.html]

Details for gene set GOMF\_OXIDOREDUCTION\_DRIVEN\_ACTIVE\_TRANSMEMBRANE\_TRANSPORTER\_ACTIVITY[GSEA]

|  || Dataset | 55457\_collapsed\_to\_symbols.cls.cls #OA\_versus\_normal.cls.cls #OA\_versus\_normal\_repos |
| Phenotype | cls.cls#OA\_versus\_normal\_repos |
| Upregulated in class | OA |
| GeneSet | GOMF\_OXIDOREDUCTION\_DRIVEN\_ACTIVE\_TRANSMEMBRANE\_TRANSPORTER\_ACTIVITY |
| Enrichment Score (ES) | 0.6845931 |
| Normalized Enrichment Score (NES) | 2.781754 |
| Nominal p-value | 0.0 |
| FDR q-value | 0.0 |
| FWER p-Value | 0.0 |
Table: GSEA Results Summary

  

Fig 1: Enrichment plot: GOMF\_OXIDOREDUCTION\_DRIVEN\_ACTIVE\_TRANSMEMBRANE\_TRANSPORTER\_ACTIVITY      
 Profile of the Running ES Score & Positions of GeneSet Members on the Rank Ordered List

  

| SYMBOL | TITLE | RANK IN GENE LIST | RANK METRIC SCORE | RUNNING ES | CORE ENRICHMENT || 1 | UQCR10 | "ubiquinol-cytochrome c reductase, complex III subunit X [Source:HGNC Symbol;Acc:HGNC:30863]" | 53 | 0.987 | 0.0375 | Yes |
| 2 | NDUFA8 | NADH:ubiquinone oxidoreductase subunit A8 [Source:HGNC Symbol;Acc:HGNC:7692] | 67 | 0.961 | 0.0771 | Yes |
| 3 | NDUFS3 | NADH:ubiquinone oxidoreductase core subunit S3 [Source:HGNC Symbol;Acc:HGNC:7710] | 177 | 0.836 | 0.1039 | Yes |
| 4 | NDUFA1 | NADH:ubiquinone oxidoreductase subunit A1 [Source:HGNC Symbol;Acc:HGNC:7683] | 194 | 0.826 | 0.1375 | Yes |
| 5 | NDUFA13 | NADH:ubiquinone oxidoreductase subunit A13 [Source:HGNC Symbol;Acc:HGNC:17194] | 236 | 0.795 | 0.1678 | Yes |
| 6 | NDUFA10 | NADH:ubiquinone oxidoreductase subunit A10 [Source:HGNC Symbol;Acc:HGNC:7684] | 243 | 0.788 | 0.2006 | Yes |
| 7 | NDUFB6 | NADH:ubiquinone oxidoreductase subunit B6 [Source:HGNC Symbol;Acc:HGNC:7701] | 262 | 0.779 | 0.2321 | Yes |
| 8 | UQCRC1 | ubiquinol-cytochrome c reductase core protein 1 [Source:HGNC Symbol;Acc:HGNC:12585] | 473 | 0.672 | 0.2441 | Yes |
| 9 | NDUFB3 | NADH:ubiquinone oxidoreductase subunit B3 [Source:HGNC Symbol;Acc:HGNC:7698] | 483 | 0.667 | 0.2715 | Yes |
| 10 | NDUFS4 | NADH:ubiquinone oxidoreductase subunit S4 [Source:HGNC Symbol;Acc:HGNC:7711] | 531 | 0.647 | 0.2951 | Yes |
| 11 | NDUFA3 | NADH:ubiquinone oxidoreductase subunit A3 [Source:HGNC Symbol;Acc:HGNC:7686] | 550 | 0.641 | 0.3208 | Yes |
| 12 | COX5B | cytochrome c oxidase subunit 5B [Source:HGNC Symbol;Acc:HGNC:2269] | 585 | 0.628 | 0.3447 | Yes |
| 13 | NDUFA4 | NDUFA4 mitochondrial complex associated [Source:HGNC Symbol;Acc:HGNC:7687] | 624 | 0.613 | 0.3676 | Yes |
| 14 | COX6A1 | cytochrome c oxidase subunit 6A1 [Source:HGNC Symbol;Acc:HGNC:2277] | 653 | 0.605 | 0.3909 | Yes |
| 15 | NDUFA2 | NADH:ubiquinone oxidoreductase subunit A2 [Source:HGNC Symbol;Acc:HGNC:7685] | 878 | 0.549 | 0.3967 | Yes |
| 16 | NDUFB5 | NADH:ubiquinone oxidoreductase subunit B5 [Source:HGNC Symbol;Acc:HGNC:7700] | 902 | 0.542 | 0.4178 | Yes |
| 17 | NDUFC2 | NADH:ubiquinone oxidoreductase subunit C2 [Source:HGNC Symbol;Acc:HGNC:7706] | 929 | 0.532 | 0.4382 | Yes |
| 18 | NDUFA9 | NADH:ubiquinone oxidoreductase subunit A9 [Source:HGNC Symbol;Acc:HGNC:7693] | 943 | 0.529 | 0.4595 | Yes |
| 19 | NDUFB4 | NADH:ubiquinone oxidoreductase subunit B4 [Source:HGNC Symbol;Acc:HGNC:7699] | 968 | 0.525 | 0.4798 | Yes |
| 20 | NDUFB7 | NADH:ubiquinone oxidoreductase subunit B7 [Source:HGNC Symbol;Acc:HGNC:7702] | 1033 | 0.510 | 0.4964 | Yes |
| 21 | NDUFB1 | NADH:ubiquinone oxidoreductase subunit B1 [Source:HGNC Symbol;Acc:HGNC:7695] | 1055 | 0.506 | 0.5161 | Yes |
| 22 | NDUFS7 | NADH:ubiquinone oxidoreductase core subunit S7 [Source:HGNC Symbol;Acc:HGNC:7714] | 1071 | 0.501 | 0.5361 | Yes |
| 23 | NDUFC1 | NADH:ubiquinone oxidoreductase subunit C1 [Source:HGNC Symbol;Acc:HGNC:7705] | 1091 | 0.499 | 0.5556 | Yes |
| 24 | NDUFA7 | NADH:ubiquinone oxidoreductase subunit A7 [Source:HGNC Symbol;Acc:HGNC:7691] | 1097 | 0.496 | 0.5762 | Yes |
| 25 | COX8A | cytochrome c oxidase subunit 8A [Source:HGNC Symbol;Acc:HGNC:2294] | 1105 | 0.494 | 0.5965 | Yes |
| 26 | NDUFS8 | NADH:ubiquinone oxidoreductase core subunit S8 [Source:HGNC Symbol;Acc:HGNC:7715] | 1208 | 0.474 | 0.6086 | Yes |
| 27 | NDUFS1 | NADH:ubiquinone oxidoreductase core subunit S1 [Source:HGNC Symbol;Acc:HGNC:7707] | 1229 | 0.471 | 0.6269 | Yes |
| 28 | NDUFS6 | NADH:ubiquinone oxidoreductase subunit S6 [Source:HGNC Symbol;Acc:HGNC:7713] | 1417 | 0.441 | 0.6310 | Yes |
| 29 | NDUFV2 | NADH:ubiquinone oxidoreductase core subunit V2 [Source:HGNC Symbol;Acc:HGNC:7717] | 1642 | 0.407 | 0.6307 | Yes |
| 30 | NDUFV1 | NADH:ubiquinone oxidoreductase core subunit V1 [Source:HGNC Symbol;Acc:HGNC:7716] | 1663 | 0.404 | 0.6462 | Yes |
| 31 | SURF1 | SURF1 cytochrome c oxidase assembly factor [Source:HGNC Symbol;Acc:HGNC:11474] | 1829 | 0.381 | 0.6494 | Yes |
| 32 | CYB561D2 | cytochrome b561 family member D2 [Source:HGNC Symbol;Acc:HGNC:30253] | 2010 | 0.360 | 0.6506 | Yes |
| 33 | COX7A1 | cytochrome c oxidase subunit 7A1 [Source:HGNC Symbol;Acc:HGNC:2287] | 2045 | 0.356 | 0.6630 | Yes |
| 34 | COX4I1 | cytochrome c oxidase subunit 4I1 [Source:HGNC Symbol;Acc:HGNC:2265] | 2167 | 0.342 | 0.6680 | Yes |
| 35 | NDUFS5 | NADH:ubiquinone oxidoreductase subunit S5 [Source:HGNC Symbol;Acc:HGNC:7712] | 2267 | 0.330 | 0.6742 | Yes |
| 36 | NDUFB2 | NADH:ubiquinone oxidoreductase subunit B2 [Source:HGNC Symbol;Acc:HGNC:7697] | 2460 | 0.307 | 0.6723 | Yes |
| 37 | COX7A2L | cytochrome c oxidase subunit 7A2 like [Source:HGNC Symbol;Acc:HGNC:2289] | 2517 | 0.302 | 0.6806 | Yes |
| 38 | NDUFB8 | NADH:ubiquinone oxidoreductase subunit B8 [Source:HGNC Symbol;Acc:HGNC:7703] | 2623 | 0.288 | 0.6846 | Yes |
| 39 | COX6B1 | cytochrome c oxidase subunit 6B1 [Source:HGNC Symbol;Acc:HGNC:2280] | 2915 | 0.259 | 0.6729 | No |
| 40 | NDUFS2 | NADH:ubiquinone oxidoreductase core subunit S2 [Source:HGNC Symbol;Acc:HGNC:7708] | 3072 | 0.243 | 0.6710 | No |
| 41 | COX7B | cytochrome c oxidase subunit 7B [Source:HGNC Symbol;Acc:HGNC:2291] | 3452 | 0.207 | 0.6502 | No |
| 42 | COX15 | cytochrome c oxidase assembly homolog COX15 [Source:HGNC Symbol;Acc:HGNC:2263] | 3727 | 0.183 | 0.6366 | No |
| 43 | UQCRH | ubiquinol-cytochrome c reductase hinge protein [Source:HGNC Symbol;Acc:HGNC:12590] | 3827 | 0.174 | 0.6362 | No |
| 44 | NDUFA6 | NADH:ubiquinone oxidoreductase subunit A6 [Source:HGNC Symbol;Acc:HGNC:7690] | 3834 | 0.173 | 0.6431 | No |
| 45 | CYC1 | cytochrome c1 [Source:HGNC Symbol;Acc:HGNC:2579] | 4879 | 0.082 | 0.5653 | No |
| 46 | COX6A2 | cytochrome c oxidase subunit 6A2 [Source:HGNC Symbol;Acc:HGNC:2279] | 5242 | 0.053 | 0.5393 | No |
| 47 | COX5A | cytochrome c oxidase subunit 5A [Source:HGNC Symbol;Acc:HGNC:2267] | 5329 | 0.045 | 0.5345 | No |
| 48 | UQCRFS1 | "ubiquinol-cytochrome c reductase, Rieske iron-sulfur polypeptide 1 [Source:HGNC Symbol;Acc:HGNC:12587]" | 5509 | 0.032 | 0.5219 | No |
| 49 | CYB5A | cytochrome b5 type A [Source:HGNC Symbol;Acc:HGNC:2570] | 5850 | 0.005 | 0.4957 | No |
| 50 | NDUFA5 | NADH:ubiquinone oxidoreductase subunit A5 [Source:HGNC Symbol;Acc:HGNC:7688] | 6789 | -0.062 | 0.4253 | No |
| 51 | CYBRD1 | cytochrome b reductase 1 [Source:HGNC Symbol;Acc:HGNC:20797] | 9585 | -0.282 | 0.2197 | No |
| 52 | MT-ND5 | mitochondrially encoded NADH:ubiquinone oxidoreductase core subunit 5 [Source:HGNC Symbol;Acc:HGNC:7461] | 12743 | -0.906 | 0.0122 | No |
Table: GSEA details [plain text format]

  

Fig 2: GOMF\_OXIDOREDUCTION\_DRIVEN\_ACTIVE\_TRANSMEMBRANE\_TRANSPORTER\_ACTIVITY      
 Blue-Pink O' Gram in the Space of the Analyzed GeneSet

  

Fig 3: GOMF\_OXIDOREDUCTION\_DRIVEN\_ACTIVE\_TRANSMEMBRANE\_TRANSPORTER\_ACTIVITY: Random ES distribution      
 Gene set null distribution of ES for **GOMF\_OXIDOREDUCTION\_DRIVEN\_ACTIVE\_TRANSMEMBRANE\_TRANSPORTER\_ACTIVITY**
